# Temporal Processing and Dichotic Listening in bilingual and non-bilingual descendants

**DOI:** 10.1016/S1808-8694(15)31040-5

**Published:** 2015-10-19

**Authors:** Raquel Mari Onoda, Liliane Desgualdo Pereira, Arnaldo Guilherme

**Affiliations:** 1 M.S. Student – Department of Speech and Hearing Therapy – Federal University of São Paulo (UNFESP-EPM).; 2 Doctor on Human Communication Disturbances, Head of the Speech Therapy Department at UNIFESP-EPM.; 3 Doctor on Otorhinolaryngology, Faculty member of the Otorhinolaryngology Department at UNIFESP-EPM. Sao Paulo Federal University - Paulista Medical School, UNIFESP/EPM.

**Keywords:** ethnic groups, multilingualism, auditory perception, hearing tests

## Abstract

The aim of this study was to analyze the auditory behavior in Pitch(PPS) and Duration(DPS)Pattern Sequence tests and in the Dichotic Listening (Dichotic Digits Test/DDT) of familiar and unfamiliar words (Staggered Spondaic Words/SSW) in Japanese descendants that speak Japanese and Japanese descendants that do not speak Japanese, and to compare these findings with a group of non-Japanese descendants who have no contact with the Japanese language. **Method:** 60 High School graduates aged 17 to 40 years were evaluated. Subjects were divided into three groups: GJJ, Japanese descendants that speak Brazilian Portuguese and Japanese; GJP, Japanese descendants that speak Portuguese and do not speak Japanese; GBP non-oriental descendants that speak Brazilian Portuguese. All subjects filled in a questionnaire about their languages and musical abilities. Their ability in pattern-recognition tests was assessed by the PPS and DPS tests, their ability to recognize familiar words was tested by DDT and their ability to recognize unfamiliar words was tested by SSW. Results. GJJ and GJP showed higher performances than the group of Brazilians (GBP) in the PPS. **Results:** show a statistically significant difference among the groups with a higher mean for the SSW results in GJJ compred to GJP and GBP. **Conclusion:**The results of SSW test seem to be influenced by bilingualism.

## INTRODUCTION

Language learning is normally done through hearing. We learn a language by listening and speaking, which then allows us to communicate ideas, feelings and desires in our environment.

There is a belief that exposure to two languages may bring benefits to auditory development.

Auditory processing is what we do with what we listen;^1^ this is a cognitive construction based on an auditory signal that makes information functionally useful. Auditory processing involves not only sound perception, but more importantly, identification, location, attention, analysis, memory, and auditory information retrieval capabilities. It is also related to the manner by which we apply previous knowledge to better understand a message and to how auditory information is integrated and associated with visual and other sensory stimuli.

There are two situations when an individual is exposed to two languages, such as Japanese and Brazilian Portuguese: one in which a greater linguistic context increases the speed and effectiveness of the information process, and the other where there is conflicting information from two different linguistic concepts, leading to processing disturbances. Thus, the issue would be whether exposure to two differently originated languages could facilitate auditory information processing, knowing that a linguistic sign has various cues including syntax, semantic, morphological and lexical cues, acoustic cues (sound pitch, intensity and duration) that lead to understanding a message.

Studies have shown a relation between learning a non-native language and anatomical and functional differences in the brain cortex of bilingual individuals.^2^, ^3^, ^4^, ^5^

Japanese has a different structure compared to Brazilian Portuguese.6 Order in Japanese syntax is: the subject (S), the object (O) and the verb (V), while the order in Brazilian Portuguese is SVO. Japanese syllables are, for the most part, composed of a vowel (V) or a consonant and a vowel (CV). Also, there are other syllables that, although not having the same syllabic structure V and CV, are equivalent to them in the time taken within an enunciate. The accent mark in Japanese characterizes sound pitch (bass x treble), different from Brazilian Portuguese, in which the stress emphasizes intensity (strong x weak).

There are structural, phonetic, writing and supra-segmental (pitch, duration, rhythm and prosody) differences between Japanese and Brazilian Portuguese. The aim of this study was to analyze the behavior of Japanese descendants living in Brazil (Japanese speakers or not) in temporal pattern recognition tests (standard pitch and duration tests) and disyllabic dichotic listening (Dichotic Digits Test and Alternated Disyllabic Dichotic Test/SSW in Portuguese), compared with the performance of a group of Brazilians not descendants of Orientals and who had no contact with the Japanese language.

## MATERIAL AND METHODS

After analysis and approval by the Research Ethics Committee of the Sao Paulo Federal University / Sao Paulo Hospital, number 1318/03, we started sample data collection. The sample included 60 male and female subjects living in Brazil, aged between 17 and 40 years, having completed at least the third year of middle school, and with no phonoaudiological complaints. Individuals that reported significant auditory difficulties and that descended from mixed oriental and western parents were excluded. We divided the sample into three groups; the first was composed of Japanese descendants who spoke both Brazilian Portuguese and Japanese (GJJ); the second group included Japanese descendants who spoke Brazilian Portuguese but not Japanese (GJP); and the third group consisted of Brazilians who were not descendants of Orientals, who spoke Brazilian Portuguese and not Japanese (GBP).

All of the subjects underwent the following tests: the Dichotic Digits Test (DDT), the Alternated Disyllable Dichotic Test / SSW (Staggered Spondaic Word) in Portuguese, the Frequency Pattern Test (FPT), and the Duration Pattern Test (DPT).

DDT and SSW are dichotic tests that simultaneously present two different speech stimuli in both ears. We used the CD version that comes together with the book “Auditory Processing Central: Manual de Avaliacao” by Pereira and Schochat (1997).^7^

FPT and DPT are time processing tests, requiring sequential temporal pattern recognition and temporal non-verbal stimuli ordering capabilities of the listener. Recognition of acoustic contours allows use to extract prosodic aspects of speech such as rhythm, stress and intonation. The individual was required to reproduce the stimulus, such as imitating (humming) and/or naming. We used the abbreviation N for naming and H for humming.

Tests were done in a silent ambience, presented to subjects through a Philips HP195 headphone model coupled to a CD player. A Lutron model SL-4001 decibelimeter was used to measure the 70 dB intensity, and the tests took approximately 45 minutes. Finally, individual answers were written in specific protocols for each test.

Brazilian and Japanese descendants were characterized through a questionnaire: identification (gender, age, schooling, generation), languages (type, fluency and degree of knowledge), and musical instrument (type and level of knowledge). This information provided a previous knowledge of the language and musical abilities of participants.

Result analysis was based on the number of right answers in each test item per group: Japanese descendants speaking Japanese (GJJ), Japanese descendants that did not speak Japanese (GJP) and Brazilians (GBP). We also reported the time pattern recognition test that subject found easiest according to their own statements.

We also used two parametric tests, ANOVA and Equality of Two Proportions. A Confidence Interval was also used to supplement descriptive analysis.

The significance level was established at 0.05 or 5%. Statistically significant values were marked with an asterisk (*), and the number sign (#) was used to show a trend towards significance.

## RESULTS

Charts I to V show the distribution of subjects according to: gender (male and female); university graduation or not; the presence of absence of musical instrument capabilities; whether second or third descendants of Japanese, as needed; and a statement finding it easier to do the FPT compared to the DPT; results were per group. The groups were GJJ or Japanese descendants speakers of Brazilian Portuguese and Japanese, GJP or descendants of Japanese speakers of Brazilian Portuguese but not Japanese, and GBP or Brazilians, not descending from Orientals, speakers of Brazilian Portuguese and non-speakers of Japanese.

**Chart I:**
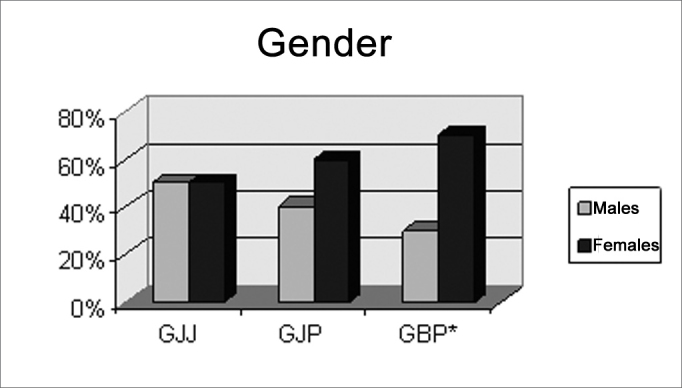
Individuals according to Gender, per pre-established group GJJ, GJP, GBP

**Chart V:**
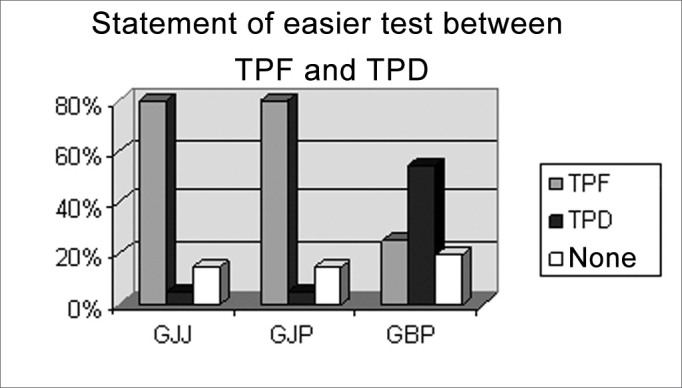
Individuals according to statement of easier test between TPF and TPD, per pre-established group GJJ, GJP, GBP.

On Table 1 we compare the variables gender (male and female), ability or not with musical instruments and complete or incomplete university training in the three groups GJJ, GJP and GBP, with stated p-values.

**Table 1:** calculated p-values used to make a comparative analysis of the performace in behavioral tests of groups GJJ,GBP, GJP considering variables: gender (male and female), skills with musical instruments and higher education.

		GJP	GJJ
Males	GJJ	0,525	
	GBP	0,507	0,197
Females	GJJ	0,525	
	GBP	0,507	0,197
Musical Instrument	GJJ	0,749	
	GBP	0,049*	0,025*
Higher education	GJJ	0,752	
	GBP	0,337	0,204

Tables 2, 3 and 4 show descriptive measurements for right answers in the behavioural tests for each of the groups GJJ, GJP and GBP. These calculations had a 95% statistical confidence interval.

**Table 2:** Descriptive measures of age range and the correct answers attained in the behavioral tests for group GJJ.

	GJJ	Average	Median	Standard Deviation	Minimum	Maximum	Size	Lower limit	Upper limit
	Age	27,45	26,5	6,69	18	40	20	24,52	30,38
TDD	OD	99,5%	100,0%	1,3%	95,0%	100,0%	20	98,9%	100,1%
	OE	99,1%	100,0%	1,2%	97,5%	100,0%	20	98,6%	99,7%
	EDD	99,1%	100,0%	2,2%	92,5%	100,0%	20	98,2%	100,1%
	EDE	99,1%	100,0%	2,0%	92,5%	100,0%	20	98,2%	100,0%
	OD	97,8%	97,5%	2,0%	92,5%	100,0%	20	96,9%	98,6%
	OE	96,6%	97,5%	3,3%	87,5%	100,0%	20	95,2%	98,1%
SSW	EO	-0,60	-0,50	1,67	-4,00	3,00	20	-1,33	0,13
	EA	-0,20	0,00	1,51	-2,00	3,00	20	-0,86	0,46
	INV.	0,25	0,00	0,44	0,00	1,00	20	0,06	0,44
TPF	Naming	91,3%	96,7%	12,3%	60,0%	100,0%	20	85,9%	96,7%
	Humming	94,8%	100,0%	9,8%	66,7%	100,0%	20	90,6%	99,1%
TPD	Naming	88,4%	91,7%	12,2%	63,4%	100,0%	20	83,0%	93,7%
	Humming	83,0%	86,7%	14,1%	56,7%	100,0%	20	76,8%	89,2%

**Legend:**

RE= right ear LE= left ear

RSH = right side directed hearing

LSH = left side directed hearing

INV = inversion AE = auditory effect OE= order effect

**Table 3:** descriptive measures of age range and correct answers attained in behavioral tests for group GJP.

	GJP	Average	Median	Standard Deviation	Minimum	Maximum	Size	Lower limit	Upper limit
	Age	23,35	22,5	4,76	17	37	20	21,26	25,44
TDD	OD	99,3%	100,0%	1,6%	95,0%	100,0%	20	98,5%	100,0%
	OE	99,1%	100,0%	1,5%	95,0%	100,0%	20	98,5%	99,8%
	EDD	99,0%	100,0%	1,9%	95,0%	100,0%	20	98,2%	99,8%
	EDE	98,4%	100,0%	3,6%	87,5%	100,0%	20	96,8%	99,9%
	OD	95,6%	97,5%	4,4%	82,5%	100,0%	20	93,7%	97,5%
	OE	93,6%	96,3%	6,0%	82,5%	100,0%	20	91,0%	96,2%
SSW	EO	-0,50	-1,00	2,26	-4,00	5,00	20	-1,49	0,49
	EA	-0,55	-0,50	1,82	-3,00	3,00	20	-1,35	0,25
	INV.	0,05	0,00	0,22	0,00	1,00	20	-0,05	0,15
TPF	Naming	93,0%	95,0%	7,8%	66,7%	100,0%	20	89,6%	96,4%
	Humming	92,7%	96,7%	8,4%	70,0%	100,0%	20	89,0%	96,4%
TPD	Naming	86,4%	90,0%	10,2%	66,7%	100,0%	20	81,9%	90,8%
	Humming	79,4%	81,7%	15,9%	36,7%	100,0%	20	72,4%	86,3%

**Legend:**

RE= right ear LE= left ear

RSH = right side directed hearing

LSH = left side directed hearing

INV = inversion AE = auditory effect OE= order effect

**Table 4:** descriptive measures of age range and correct answers attained in behavioral tests for group GBP.

	GBP	Average	Median	Standard Deviation	Minimum	Maximum	Size	Lower limit	Upper limit
	Age	22,65	22	2,91	17	30	20	21,38	23,92
TDD	OD	99,4%	100,0%	1,1%	97,5%	100,0%	20	98,9%	99,9%
	OE	99,5%	100,0%	1,3%	95,0%	100,0%	20	98,9%	100,1%
	EDD	99,5%	100,0%	1,5%	95,0%	100,0%	20	98,8%	100,2%
	EDE	99,0%	100,0%	1,5%	95,0%	100,0%	20	98,3%	99,7%
	OD	94,9%	97,5%	4,5%	85,0%	100,0%	20	92,9%	96,8%
	OE	94,4%	96,3%	5,6%	80,0%	100,0%	20	91,9%	96,8%
SSW	EO	-0,05	0,00	1,96	-3,00	5,00	20	-0,91	0,81
	EA	-0,50	0,00	1,91	-5,00	3,00	20	-1,34	0,34
	INV.	1,50	0,50	3,46	0,00	15,00	20	-0,01	3,01
TPF	Naming	80,2%	83,3%	19,3%	46,7%	100,0%	20	71,7%	88,6%
	Humming	80,8%	90,0%	20,8%	40,0%	100,0%	20	71,7%	90,0%
TPD	Naming	85,2%	88,4%	15,6%	43,4%	100,0%	20	78,3%	92,0%
	Humming	80,3%	83,3%	16,0%	40,0%	100,0%	20	73,3%	87,4%

**Legend:**

RE= right ear LE= left ear

RSH = right side directed hearing LSH = left side directed hearing

INV = inversion AE = auditory effect OE= order effect

Table 5 shows statistical test values done to compare right answers by ear, right ear (RE) and left ear (LE) in dichotic procedures (DDT and SSW), right directed listening (EDD) and left directed listening (EDE) in the DDT, per type of answer, humming (H) and naming (N), and average number of right answers in temporal pattern recognition procedures (FPT and DPT).

**Table 5:** calculated p-values used to compare right answers for the right ear (RE) and the left ear (LE) in dichotic procedures (SSW and TDD), on the stages of free attention and right side directed hearing (RSH) and left side directed hearing (LSH) in TDD, and by type of response (humming or naming) and average of right answers in the temporal standard recognition procedures ( TPF and TPD) per group

	TDD		SSW	TPF	TPD	TPF x TPD
	Free attention	Directed hearing				
	RE X LE	RSH X LSH	RE X LE	N X H	N X H	
GJJ	0,355	1,00	0,196	0,325	0,209	0,08*
GJP	0,801	0,492	0,235	0,912	0,106	< 0,001*
GBP	0,746	0,304	0,757	0,917	0,339	0,576

**Legend:**

RE= right ear LE= left ear

N = naming H = humming

Table 6 shows the statistical test values done to compare right answers in the various dichotic listening procedures, regardless of which ear, and pattern recognition, regardless of the type of response, in the three groups (GJJ, GJP and GBP).

**Table 6:** calculated p-values used to compare right answers in dichotic procedures (TDD and SSW) and standard recognition (TPF and TPD) among groups GJJ, GJP and GBP.

	GJJ X GJP X GBP	
TDD – EDD	0,708	
TDD – EDE	0,492	
SSW	0,015*	
TPF	< 0,001*	
TPD	0,584	
	p-value used to compare the groups for SSW	
	GJJ	GJP
GJP	0,008*	
GBP	0,006*	1,00
	p-value used to compare the groups for TPF	
	GJJ	GJP
GJP	0,918	
GBP	0,001*	< 0,001*

## DISCUSSION

Bilingual children can respond using either language according to the language used by the speaker. This may result from a metalinguistic competency obtained early in life.^8^

A variety of mechanisms may be used to process language. SSW is an auditory processing test which demands linguistic competency.^9^

Studies in international literature suggest a close relationship between learning different languages and anatomical, morphological and behavioral changes in the brain.^2^, ^3^, ^4^, ^5^, ^10^

The acoustic difference between phonemes in Portuguese is larger than in English, which on the other hand has more complex phonetics. Thus, one may think that Portuguese language phonetics provides better training in duration resolution than frequency.^11^ We found no published paper comparing Japanese with Brazilian Portuguese on the acoustic perception of speech sounds.

Tone patterns are recognized as music or melody, being composed of tones with different frequencies and duration in various time sequences. Furthermore, tone patterns depend on a variety of central auditory processes, including recognition as a whole, inter-hemispheric transference, linguistic naming, sequencing of logistic elements and memory.^12^

Music and language establish semantic processing physiological indices, in which the word meaning is the main element, both in language and in music.^13^

Individuals who are unable to recognize time patterns cannot extract and use prosodic aspects of speech such as rhythm, stress and tone, which allow the listener to identify key words within a sentence and to interpret emphasis and irony. Such individuals may be incapable of discriminating subtle differences in meaning just based on changes in stress or tone.^14^

We start our specific discussion on an analysis of the characteristics of each group.

There was no statistically significant difference in gender, schooling and abilities with musical instruments in GJJ and GJP (Charts I, II, III), showing homogeneity in these groups.

**Chart II:**
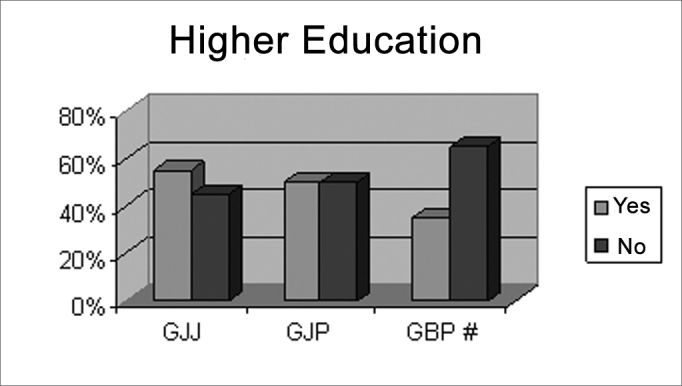
Individuals according to higher education or not, per pre-established group GJJ, GJP, GBP.

**Chart III:**
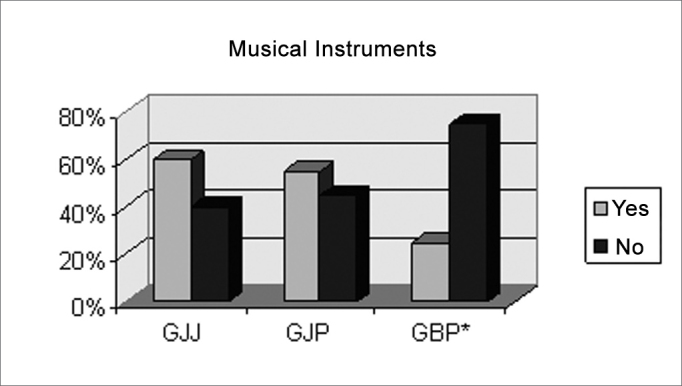
Individuals according to having musical instruments skills or not, per pre-established groups GJJ, GJP, GBP.

We found that GJJ included mostly second and third generation Japanese descendants, and that 95% of the GJP group was composed of third generation Japanese; these numbers are statistically significant (Chart IV). We may state that there is an influence of the Japanese culture in both GJJ and GJP, as most of the individuals in these groups are children or grandchildren of Japanese immigrants.

**Chart IV:**
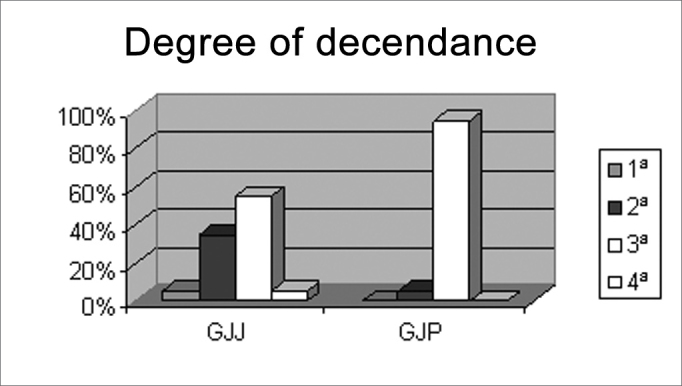
Individuals according to the degree of decendance for groups GJJ and GJP

The FPT was considered the easiest exam by 80% of individuals in both groups GJJ and GJP (Chart V).

There was a statistically significant difference in gender and ability with musical instruments in group GBP (Chart I and III).

There is a trend towards a difference in university schooling, as the p-value is very close to the acceptable limit (Chart II); this is probably due to the fact that most individuals are still undergraduate students.

We were unable to define which was the easiest test between FPT and DPT; although DPT recurred proportionally with greater frequently, it is not possible to state that it is different from the proportion of FPT. There is a trend in this direction, though (Chart V).

We also found that there are more women than men in group GBP. Few members of this group completed university studies or had abilities with musical instruments.

There is a proportional distribution in each group when comparing the variables gender and a completed university undergraduate course (Table 1), suggesting that these variables were well adjusted. Most of the individuals in groups GJJ and GJP were able to play some musical instrument, different from group GBP (Table 1). GBP shows a proportionally significant difference compared to the other groups in playing or not a musical instrument.

The following analysis looks at the number of right answers for each test in each group (Tables 2, 3 and 4). There was a low variation in the number of right answers in dichotic tests (DDT and SSW) for all groups, with every individual performing similarly. There was a higher variation in the time processing tests (FPT and DPT), with different performances within each group.

The DDT compared the number of right answers for the RE and LE and the directed listening for the right ear (EDD) and the left ear (EDE). The SSW test compared the RE and the LE, and the FPT and DPT compared right answers in each type of answer (humming and naming). There were no statistically significant differences between the groups in these variables. We also compared the average right response number for the FPT and DPT in each group (Table 5). There was a statistically significant difference between groups GJJ and GJP. This finding shows that Japanese descendants, speakers or not of Japanese, had a higher performance in time ordering of a frequency pattern compared to the time ordering of a sound duration pattern.

On the FPT there is a difference in the average number of right answers that was statistically significant for all groups. GBP was different from the other groups.

There was no significant difference in the average number of right answers in the DDT for all groups (Table 6). This finding suggests that the DDT is not affected by knowledge of languages or musical abilities. Average values are above those reported in some papers that found percentages of right answers of 90 and 97.8%.^20^, ^21^, ^22^

The first Brazilian study on children, teenagers and adults using DDT (the same version used in this study) showed a RE advantage in children and teenagers but not in adults.^21^ The same test applied to adults and elderly individuals in another Brazilian study also reported a RE advantage.^23^ Our findings using DDT did not show any RE advantage.

There is a statistically significant difference for the average number of right answers between the groups using the SSW test (Table 6). The group GJJ was different, with a higher average number of right answers compared to the other groups (Table 2). This finding suggests that Japanese descendants who speak Japanese have a higher performance in the figure-background auditory ability. Further studies are needed to clarify this finding.

There was a significant difference in the FPT between the number of right answers obtained by the various groups (Table 6). GBP was the different group (80.2% on naming and 80.8% on humming), with a significantly lower average compared to the other groups (Table 4). GJJ was the group with the highest average (91.3% on naming and 94.8% on humming), but this difference was not statistically different from GJP (93% on naming and 92.7 on humming). This finding suggests that Japanese descendants, speakers or not of Japanese, had a higher average number of right answers compared to Brazilians on time ordering of a sound frequency pattern.

There was no statistically significant difference among groups in the DPT averages (Table 6), suggesting that knowledge of different languages has no influence on this test and abilities with musical instruments does not affect the ability for auditory time ordering of a sound duration pattern.

Results were compared with findings in specialized literature on audiologically and neurologically normal individuals. We point out that to date there are no published Brazilian studies relating auditory processing using Japanese language.

There was no statistically significant difference in group averages on the DDT and DPT. This suggests that such tests may be used for screening purposes in the assessment of auditory processing, particularly in foreign and/or bilingual individuals and persons with musical abilities, as knowledge of Japanese and musical abilities did not affect the results.

A study done on Brazilian adults showed that the DPT performance was better than FPT performance. Subjects reported that concepts such as “long,” “short” and “time duration” were more easily perceived. The author of this paper states that the acoustic nature of Brazilian Portuguese probably facilitates performance in recognizing the duration pattern studied.^15^

We found that the DPT performance was similar to the FPT performance in the Brazilian group. However, it was lower in the Japanese descendant groups. Thus, we believe that the auditory nature of each language may interfere on auditory processing behavioural tests, even for non-verbal stimuli.

The GJJ group showed a significantly higher performance on the SSW test in Portuguese compared to other groups. Results were similar to findings in specialized literature, which shows average values equal to or below two.^24^, ^25^

Therefore, we hypothesize that the structure and nature of Japanese, together with cultural influences, may influence figure-background auditory abilities for low predictability disyllabic words in a dichotic task. We found no published paper reporting the performance of bilingual individuals speaking Japanese and Brazilian Portuguese in auditory processing tests. Studies on learning and/or exposure to two different languages show that there are anatomical, morphological and behavioural alterations in the brain in these situations.^2^, ^3^, ^4^, ^5^, ^10^. This may be related to the fact that the group of Japanese descendants who spoke Brazilian Portuguese and Japanese had a significantly superior performance compared to the other groups.

For the FPT, groups GJJ and GJP showed a statistically superior performance compared to the GBP group. We may attribute this to the influence of the Japanese language, as stresses in this language are mostly on tone pitch (bass x treble) different from Portuguese, where stress is mostly on intensity (strong x weak).6 Furthermore, second and third generation descendants of Japanese still use occasional Japanese words daily, even though they might not be fluent in the language.

Another point was the increased ability with musical instruments seen in groups GJJ and GJP compared to group GBP. Some papers have suggested that duration patterns are processed differently from frequency (pitch) and intensity patterns.16,17 Differences in auditory processing on frequency pattern tests in musicians and non-musicians may result from musical training. Furthermore, the latter depend more on important brain regions to discriminate frequency patterns, which the former use specialized regions for short-term memory for improved performance in frequency memory tasks.18 Frequency discrimination is influenced by specific phenomena in music instrument training.^19^

In this study we applied FPT and DPT stimuli binaurally, that is, the same stimulus was simultaneously presented to both ears. Studies have shown that there is no difference in right and left ear performance in both tests.^11^, ^15^, ^26^, ^27^ Both cerebral hemispheres, each with a specific function but working jointly, regardless of the stimulated ear, are involved in the temporal sequencing tasks required in auditory pattern tone tests. Structures involved in auditory pattern tone tests are the hemispheres and the corpus callosum, the structure that connects both. The right hemisphere is activated by global pattern recognition (gestalt) and the left hemisphere is responsible for ordering the sequence of stimuli and naming what was heard.^28^

There was no significant difference in the type of FPT and DPT response (humming x naming) in the three groups. A recent study found similar results.^29^

In the FPT we found approximate average values of 81% for humming or naming in group GBP individuals. This value is roughly 10% below the average number of right answers in GJJ and GJP individuals. These differences were statistically significant.

In the DPT we found a similar number of average right answers: 85.7% (GJJ), 82.9% (GJP) and 82.8% (GBP). The average for all groups in the FPT and the DPT was above the average in published literature and over the reference cut off points recommended in some studies.^11^, ^15^, ^26^

Groups GJJ and GJP had a significantly higher average number of right answers in the FPT compared to the DPT, which is similar to internationally published results12 and different from Brazilian published papers.^11^, ^15^, ^27^, ^29^ This difference between the FPT and the DPT raise the hypothesis that Portuguese language phonetics might provide better training for duration than frequency resolution, as the acoustic differences between phonemes in this language are larger compared to English (which has more complex phonetics than Portuguese). Another possible hypothesis is the fact that children and teenagers living in Brazil are not habitually taught music - music is not a compulsory subject at school - different from the North American population and the GJJ and GJP groups in our study, most of which have some kind of ability with musical instruments. Thus, pure tone frequency and duration pattern recognition may be influenced by musical experience.

## CONCLUSION

Based on our results we may conclude for these groups that:

- Japanese descendants speaking or not speaking Japanese have a higher rate of right answers on the FPT compared to the group of Brazilians, and a better performance on the FPT compared to the DPT. There was no difference in the average number of right answers in the DPT for the three groups. Therefore, it appears that auditory experience provided by musical instruments and/or experience with the Japanese language facilitated frequency pattern recognition for the sound frequency we studied.
- The performance in dichotic listening of low predictability syllables, which is the SSW test in Portuguese, is positively influenced by bilingualism, as Japanese descendants, speakers of Japanese, had a significantly higher rate of right answers on the SSW test. The dichotic listening of highly predictable disyllables, which is the DDT in Portuguese, appears not to be affected by bilingualism (Japanese and Brazilian Portuguese), as there was no significant difference in the average number of right answers for the three groups.
